# An Anti-Ubiquitin Antibody Response in Transitional Cell Carcinoma of the Urinary Bladder

**DOI:** 10.1371/journal.pone.0118646

**Published:** 2015-03-05

**Authors:** Peter U. Ardelt, Jan Ebbing, Fabian Adams, Cora Reiss, Wadih Arap, Renata Pasqualini, Alexander Bachmann, Ulrich Wetterauer, Hubertus Riedmiller, Burkhard Kneitz

**Affiliations:** 1 Department of Urology, University Hospital Basel, Basel, Switzerland; 2 Department of Urology and Pediatric Urology, Medical School, Albert-Ludwigs-University of Freiburg, Freiburg, Germany; 3 Department of Urology, Bavarian Julius Maximilians-University Medical School, Würzburg, Germany; 4 Center for Thrombosis and Hemostasis, Johannes Gutenberg University Medical Center Mainz, Mainz, Germany; 5 Division of Hematology/Oncology and Division of Molecular Medicine, Department of Internal Medicine, University of New Mexico School of Medicine, Albuquerque, New Mexico, United States of America; Taipei Medicine University, TAIWAN

## Abstract

**Background:**

To use combinatorial epitope mapping (“fingerprinting”) of the antibody response to identify targets of the humoral immune response in patients with transitional cell carcinoma (TCC) of the bladder.

**Methods:**

A combinatorial random peptide library was screened on the circulating pool of immunoglobulins purified from an index patient with a high risk TCC (pTa high grade plus carcinoma in situ) to identify corresponding target antigens. A patient cohort was investigated for antibody titers against ubiquitin.

**Results:**

We selected, isolated, and validated an immunogenic peptide motif from ubiquitin as a dominant epitope of the humoral response. Patients with TCC had significantly higher antibody titers against ubiquitin than healthy donors (p<0.007), prostate cancer patients (p<0.0007), and all patients without TCC taken together (p<0.0001). Titers from superficial tumors were not significantly different from muscle invasive tumors (p = 0.0929). For antibody response against ubiquitin, sensitivity for detection of TCC was 0.44, specificity 0.96, positive predictive value 0.96 and negative predictive value 0.41. No significant titer changes were observed during the standard BCG induction immunotherapy.

**Conclusions:**

This is the first report to demonstrate an anti-ubiquitin antibody response in patients with TCC. Although sensitivity of antibody production was low, a high specificity and positive predictive value make ubiquitin an interesting candidate for further diagnostic and possibly immune modulating studies.

## Introduction

Transitional cell carcinoma (TCC) of the urinary bladder is among the most common genitourinary cancer. It is the fourth and ninth most common cancer in men and women, respectively, in the Western world, although incidence has been decreasing over the last decades in some countries, presumably due to the decrease in tobacco use [1]. Mortality strongly depends on grading and clinical staging. In TCC a broad bandwidth exists for the potential to recur or progress ranging from low (e.g. G1/low grade disease) to high aggressiveness (e.g. G3/high grade disease). Most commonly diagnosis is made after gross hematuria (70%). Once diagnosed, about 70% of TCC are classified as superficial and treated by transurethral resection in combination with adjuvant chemo- or immunotherapy [1, 2]. Overall, almost 69–90% of TCC will recur after transurethral resection alone [3, 4]. Therefore, a prudent follow up examination scheme is required. Costs for current treatments and follow up procedures are high, making TCC currently socio-economically the most expensive tumor entity [5].

As well as being expensive, the diagnosis and follow-up of TCC, which currently rely on cystoscopy and urine cytology, are far from ideal. Cystoscopy is an invasive and unpleasant procedure, which has led to their low acceptance in patients [6, 7]. In addition, cystoscopy has a tendency to miss flat lesions, such as carcinoma in situ, while urine cytology is prone to missing well differentiated low grade lesions [2, 8]. Furthermore, both methods are dependent on observer expertise. Therefore large efforts have been undertaken to develop alternative approaches for the diagnosis and follow up of TCC [9, 10].

In the last decades a large number of diagnostic markers, mostly urine based, have been published, e.g. nuclear matrix protein (nmp) 22 or bladder tumor associated antigen (BTA) stat, only to be rejected in critical reviews shortly thereafter [8]. Although sensitivity of these biomarkers is often high, specificity is low resulting in unnecessary cystoscopies and biopsies, which are highly disturbing for patients [8]. Therefore, there is an urgent need to identify new and robust biological markers for TCC.

The Ubiquitin Proteasome System (UPS) regulates intracellular protein homeostasis

by degrading excess, mutated or misfolded proteins by poly-ubiquitination and successive cleavage by the proteasome [11]. These protein fragments are then degraded to recycle ubiquitin and enable either single amino acids or smaller fragments to be presented by the major histocompatibility complex (MHC). Upon cellular stress, such as lack of oxygen in quickly proliferating tumors, an increased amount of ubiqitinated proteins accumulates in the cell. Such an abnormal accumulation of ubiquitin or ubiquitinated proteins has been described in Parkinson’s disease, Alzheimer’s disease, as well as cancer such as chronic B cell lymphoma [12–14].

Given that the immune response has an established role as a predictive biomarker in cancer therapy of many tumors including human bladder cancer we hypothesized that a serum-based biomarker for diagnosis of TCC could be identified using combinatorial epitope mapping (“fingerprinting”) of the antibody response [15–18],

## Materials and Methods

### Collection of Patient Serum Samples

All experiments were reviewed and approved by the Institutional Review Board (IRB) of the University of Freiburg, Germany and registered with the German Clinical Trials Register (www.drks.de; DRKS00003700). This project was performed in strict accordance with the Declaration of Helsinki. Ethical considerations regarding the use of animals for the generation of a polyclonal rabbit serum have been taken into account as required by European and national statutory provisions and in accordance with those set forth by the *Guide for the Care and Use of Laboratory Animals* (National Research Council of the National Academies).‬‬‬‬‬‬‬‬‬‬‬‬‬‬‬‬‬‬‬‬‬‬‬‬‬‬‬‬‬‬‬‬‬‬

A written informed consent was obtained from all patients. Serum samples were collected and immediately frozen at -80°C after centrifugation from patients undergoing transurethral resection for suspected TCC or undergoing cystectomy. Blood samples of TCC patients were collected a maximum 1 week before and latest one day before surgery. In case of patients undergoing immunotherapy with Mycobacterium bovis Bacillus-Calmette-Guerin (BCG) blood samples were taken 1 day before start of the induction therapy and 6 weeks after last BCG application (3 months after start). Control samples were from patients with prostate cancer before operation and age-matched healthy volunteers. Standard exclusion criteria were any autoimmune disease, immune deficiency and other tumors.

### Screening of the Phage Display Random Peptide Library

A random peptide phage display library based on the vector fUSE5 with the general arrangement CX_7_C (C, cysteine; X, any residue) and insertless phage clones as negative control were used [19]. Patients IgGs were immobilized and/or purified on protein G-agarose beads for all experiments (Sigma-Aldrich). The screening was performed as described elsewhere [19]. In brief, 10^9^ transducing units (TU) of the CX_7_C random peptide phage display library were pre-cleared on pooled immobilized IgG from healthy control volunteers for 60 min at 4°C. After centrifugation, equal amounts of the supernatant were added to immobilized IgGs from an index bladder cancer patient and healthy control volunteers. After incubation for 2 h at 4°C, beads were washed 5 times with phosphate buffered saline (PBS). Bound phage were eluted with 0.1 M glycine buffer (pH = 2.2) and neutralized with 1 M Tris/HCl (pH = 7.0). Re-infection was performed with 1 ml of log-phase *E*. *coli* K91. Sequential rounds of phage amplification were performed as described [19] and serial dilutions plated in triplicates to determine the ratio of bound phage after each screening round.

### DNA Amplification and Sequencing

Starting at the second selection round, serial dilutions were plated for single bacterial colony isolation. Individual DNA corresponding to the peptide inserts of randomly picked phage clones (n = 35) was amplified with the primers 5′-AGGTTGGTGCCTTCGTAGTG-3′ and 5′-GTTTAGTACCGCCACCCTCA-3′. After purification with EXO SAP IT, (USB, Cleveland, Ohio, USA) sequencing was performed with the primer 5′-CCCTCATAGTTAGCGTAACGATCT-3′, the CEQ2000 Dye Terminator Cycle Sequencing Kit, and a CEQ 2000 automated sequencer (Beckmann Coulter, Fullerton, CA). Sequences were aligned and compared to online protein databases of the National Center for Biotechnology Information (http://ncbi. nlm.nih.gov/blast/Blast.cgi).

### Generation of GPGARPI-GST- fusion protein and polyclonal rabbit antiserum

Next, a gluthatione-S-transferase (GST)-fusion protein with the sequence GPGRAPI was cloned, using the vector pGEX2TK, as described elsewhere [20]. Wildtype-GST served as a negative control. A polyclonal rabbit antiserum was commercially generated (immunoGlobe, Himmelstadt, Germany) with GPGRAPI-GST-fusion protein. Rabbit serum samples were obtained prior to pre-bleed and after repeated immunization. Binding affinity and specificity were verified in a binding and a binding inhibition assay using pre-immune and immunized serum on GPGRAPI-GST fusion and a control GST peptide.

### Phage Binding Assay and Competitive Binding Inhibition

For phage binding assays, 1 μg of purified IgG was immobilized on Nunc Maxisorp plates (Nunc, Wiesbaden, Germany) overnight at 4°C. After blocking wells with PBS containing 0.1% Tween-20 and 1% non fat dry milk (NFDM) for 2h at room temperature (RT), we added 10^8^ TU of either amplified phage clones or control phage for 2h at RT. For inhibition assays, increasing amounts of corresponding GST-fused peptides or wild-type (negative control) GST were pre-incubated for 30 min at RT prior to addition of phage. Unbound phage were removed by washing 5 times with PBS containing 0.1% Tween-20 and 1% NFDM. Bound phage were recovered with 200 μl log-phase *E*. *coli* K91 and plated in serial dilutions (triplicate plates for each dilution).

### Cell culture and fractionization

The TCC cell line T24 was grown (American Type Culture Collection, Anassas, Virginia, USA) to 70% confluence [21], harvested with 2,5mM EDTA, pelleted and resuspended in TM buffer (250mM Sucrose, 0.01M Tris-HCl, pH 7.4, 0.002M MgCl, 1% Triton X100, 10μg/ml Aprotinin, 10μg/ml Leupeptin). After passing the solution 10 times through a 22Gauge needle, non lysed cells were removed by centrifugation (1000g, 5min). The nuclear fraction was then pelleted by centrifugation (2000g, 10min) and resuspended in NL buffer (0.5% Triton X100, 0.1% SDS, 50mM Tris pH 7.4, 10mM NaCl, 10μg/ml Aprotinin, 10μg/ml Leupeptin). The supernatant was then centifuged for 10min at 8000g and the pellet containing the cytosolic proteins and mitochondria resuspended in NL buffer.

### Western Blot and MALDI analysis

150 μl of cytosolic and nuclear fraction of T24 tumor cells were resolved by electrophoresis on a 12.5% SDS-polyacrylamide gel and proteins electrotransferred onto a polyvinylidene fluoride (PVDF) membrane. After blocking the membrane with PBS containing 7% NFDM, 100 μl of the polyclonal anti-rabbit serum (1:200) was added at 4°C overnight. Pre-immune serum from the same rabbit was used as a negative control. After washing with PBS containing 7% powder milk, bound IgGs were detected with a peroxidase coupled swine-anti-rabbit (1:4000) secondary antibody (Sigma-Aldrich). Detection was performed with the Roti-Lumin reagent (Roth, Karlsruhe, Germany).

For MALDI (matrix-assisted laser desorption/ionization) analysis bands detected bands at 55 and 22kD were excised from the native gel by comparison to the blot and sent for commercial MALDI analysis (ProtTech, Phoenixville, Pensylvania, USA).

### ELISA and Patient Screening

For enzyme-linked immunosorbent assay (ELISA), 1 μg of purified glyceraldehyde 3-phosphate dehydrogenase (GAP-DH; Abcam, Cambridge, UK) or purified Ubiquitin (Boston Biochem, Cambridge, Massachusetts, USA) per well was immobilized on Maxisorp plates (Nunc) overnight at 4°C. Wells were blocked with PBS containing 0.1% Tween-20 and 1% NFDM for 2 h at RT. Next, 100 μl of 1:200 diluted serum was added overnight at 4°C. After 5 washes with PBS containing 0.5% NFDM, bound antibodies were detected with peroxidase-coupled rabbit-anti-human-IgG (diluted 1:5000) or goat-anti-human-IgA secondary antibody (1:5000) for 1 h at RT (both Sigma-Aldrich). The substrate tetramethylbenzidine (Sigma-Aldrich) was added for 20 min and absorbance at 620 nm was determined in an automated ELISA plate reader (LabSystems, Finland). For competitive inhibition, serum samples were pre-incubated with GPGRAPI-GST-fusion protein or synthetic GPGRAPI peptide (CGPGRAPIC; Life Technologies, Grand Island, NY, USA) for 20 min at RT. Wildtype-GST or a scrambled synthetic peptide (RPIGGAP; Life Technologies, USA) were used as negative controls, respectively.

### Biostatistics

Optical densities of antibody reaction measured in ELISAs and phage binding were compared with two-sided Student t-tests (Mann-Whitney U Test). For all statistics calculations, graphpad prism 6 was used.

## Results

### Combinatorial Screening on IgG from a Bladder Cancer Patient

**Fig 1:**
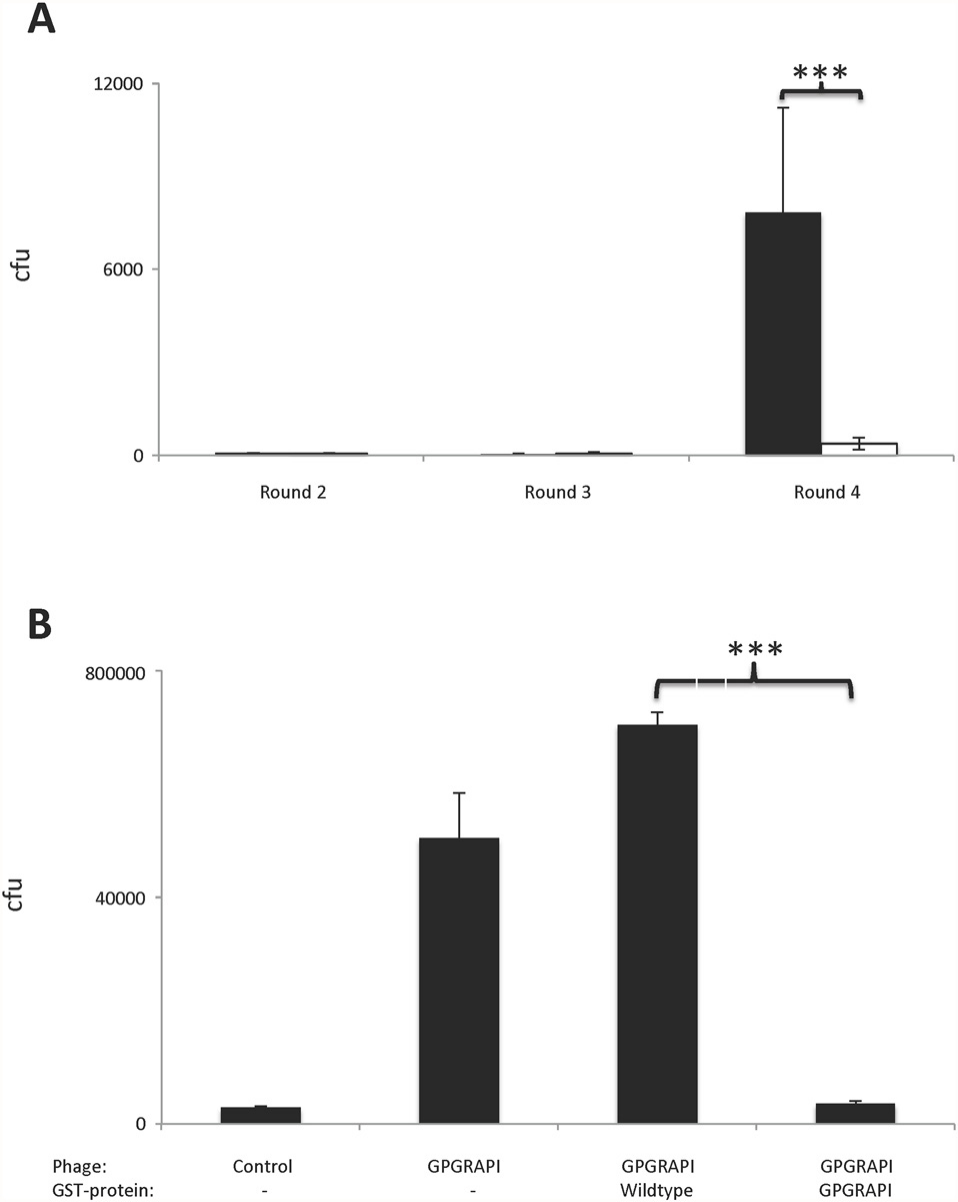
Screening of a random peptide phage display library on immobilized IgG from the index patient. A) Continuous enrichment of selected phage during the biopanning (Black column selected phage, white column control phage). B) Selected and enriched phage GPGRAPI bind specifically to immobilized IgG: Addition of GPGRAPI-GST but not wildtype-GST fusion protein competitively inhibits phage binding (p<0.0001) to immobilized IgG of the index patient. Control phage bind lower than GPGRAPI phage. Error bars represent standard deviation of triplicates, *** indicates p <0.0001. cfu = colony forming units.

For screening purposes an index patient with a high risk bladder cancer (Carcinoma in situ plus papillary high grade TCC) was chosen. The circulating antibody pool was probed with a random peptide phage display CX_7_C library. A continuous enrichment of selected phage clones during the biopanning was observed in contrast to an insertless phage clone (Fig. 1A; p<0.001). This indicates a preferred binding of the selected phage clones to the IgG of the index patient.

Subsequent DNA sequencing revealed a predominant motif GPGRAPI found in 7 out of 35 sequenced clones. Three further peptide motifs similar to GPGRAPI with the dominant shared epitope of GRAXI were identified in a total of 12 other phage clones (Table 1).

**Table 1:** 

Selected phage clones									
G	P	G	R	A	P	I			(7x)
		G	R	A	S	I	W	P	(1x)
G	M	G	R	E	S	A			(6x)
	V	G	R	R	P	W	F		(5x)

### Verification of GPGRAPI binding specificity

The binding of amplified GPGRAPI-phage to the IgG of the index patient was higher than an insertless phage (Fig. 1B). Furthermore, the binding of this sequence was specific: binding of GPGRAPI displaying phage was competitively inhibited by addition of GPGRAPI-GST but not wildtype-GST fusion protein (p<0.0001) (Fig. 1B).

### Generation and validation of a polyclonal rabbit anti GPGRAPI serum

**Fig 2:**
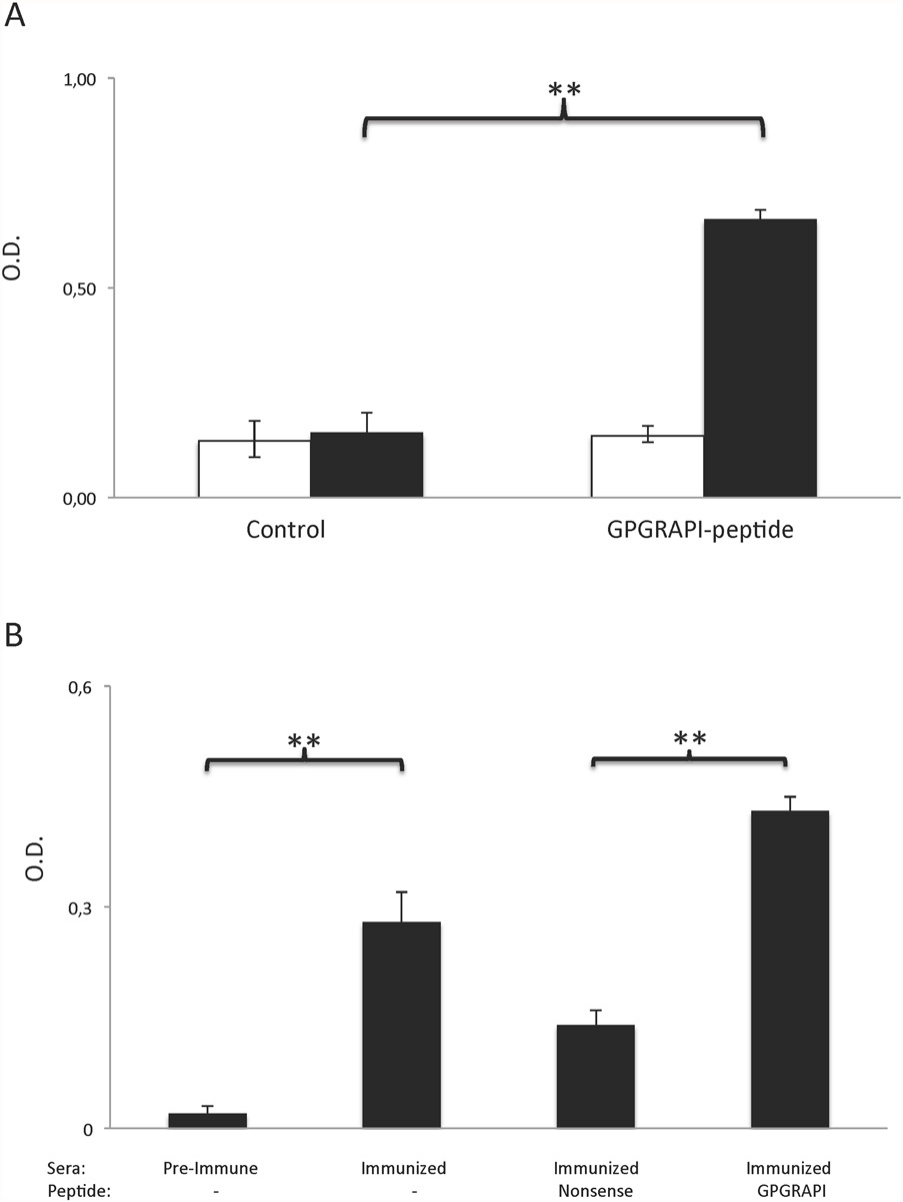
Polyclonal rabbit anti GPGRAPI-GST serum: A) After immunization the polyclonal antiserum but not a control protein (scrambled sequence) strongly binds to immobilized synthetic GPGRAPI. Pre-immune rabbit serum (white column) served as negative control and did not bind to either peptides. B) Binding of the polyclonal antiserum to immobilized GPGRAPI-GST fusion protein is competitively inhibited by synthetic GPGRAPI- but not by the control peptide. Preimmune serum from the same rabbit served as negative control. Error bars represent standard deviation of triplicates, ** indicates p <0.001

A polyclonal rabbit anti GPGRAPI-GST serum was generated commercially. After immunization of the rabbit the antiserum strongly bound to immobilized GPGRAPI—GST fusion protein (Fig. 2A). Pre-immune serum served as a negative control. This binding proved specific as binding of the polyclonal antiserum but not of the pre-immunization serum to immobilized GPGRAPI-phage was competitively inhibited by the GPGRAPI-GST fusion protein (Fig. 2B).

### Identification of the GPGRAPI corresponding target antigen

**Fig 3:**
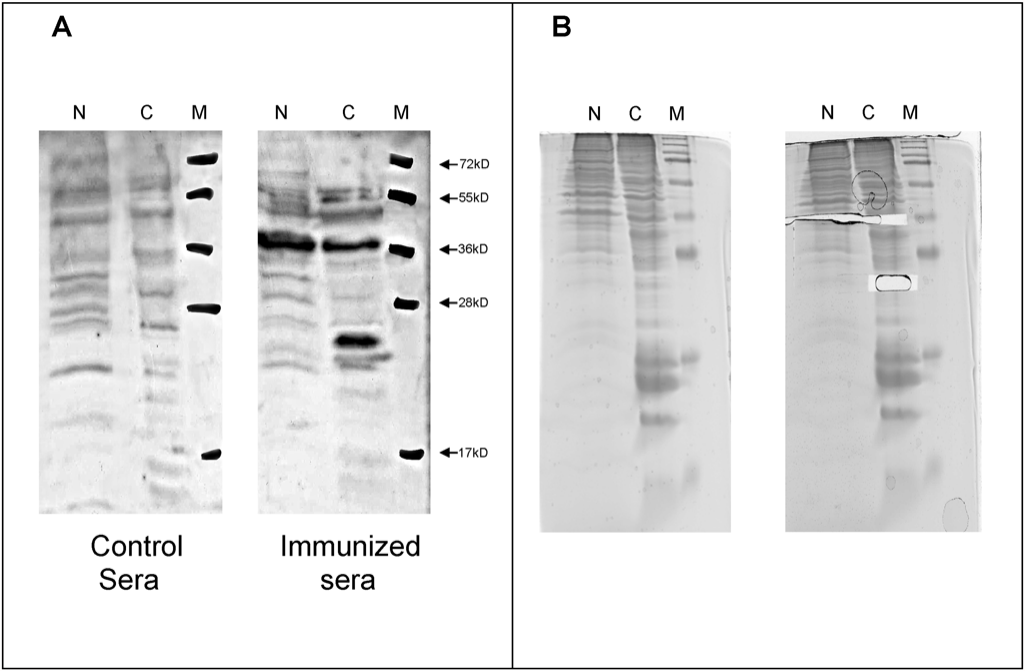
Identification of the corresponding antigen to the peptide sequence GPGRAPI. Protein isolates of the human urothelial cancer bladder cell line T24 was harvested, lysed and fractionated. (N = nuclear, C = cytosolic fraction, M = protein marker). A) Western Blot, stained with preimmune (left gel) and the immunized (right gel) rabbit serum. B) Corresponding gel before (left gel) and after excision (right gel) of the recognized bands for MALDI analysis.

To identify the corresponding target antigen containing the peptide sequence GPGRAPI we fractionated and resolved protein isolates of the human urothelial cancer bladder cell line T24 by Western blot (Fig. 3A). The polyclonal rabbit anti GPGRAPI-GST serum detected a 36kD band in both the nuclear and cytosolic fraction as well as a 22kD band in the cytosolic fraction. These bands were excised from the gel and analyzed by mass spectrometry (Fig. 3B).

MALDI analysis of the 22kD band revealed poly-ubiquitin as the dominant content (52.2%). Other proteins present were of minor content (7.2% ribosomal protein S7 and 6.1% ribosomal protein S9 as well as 20 further proteins each below 5%).

**Fig 4:**
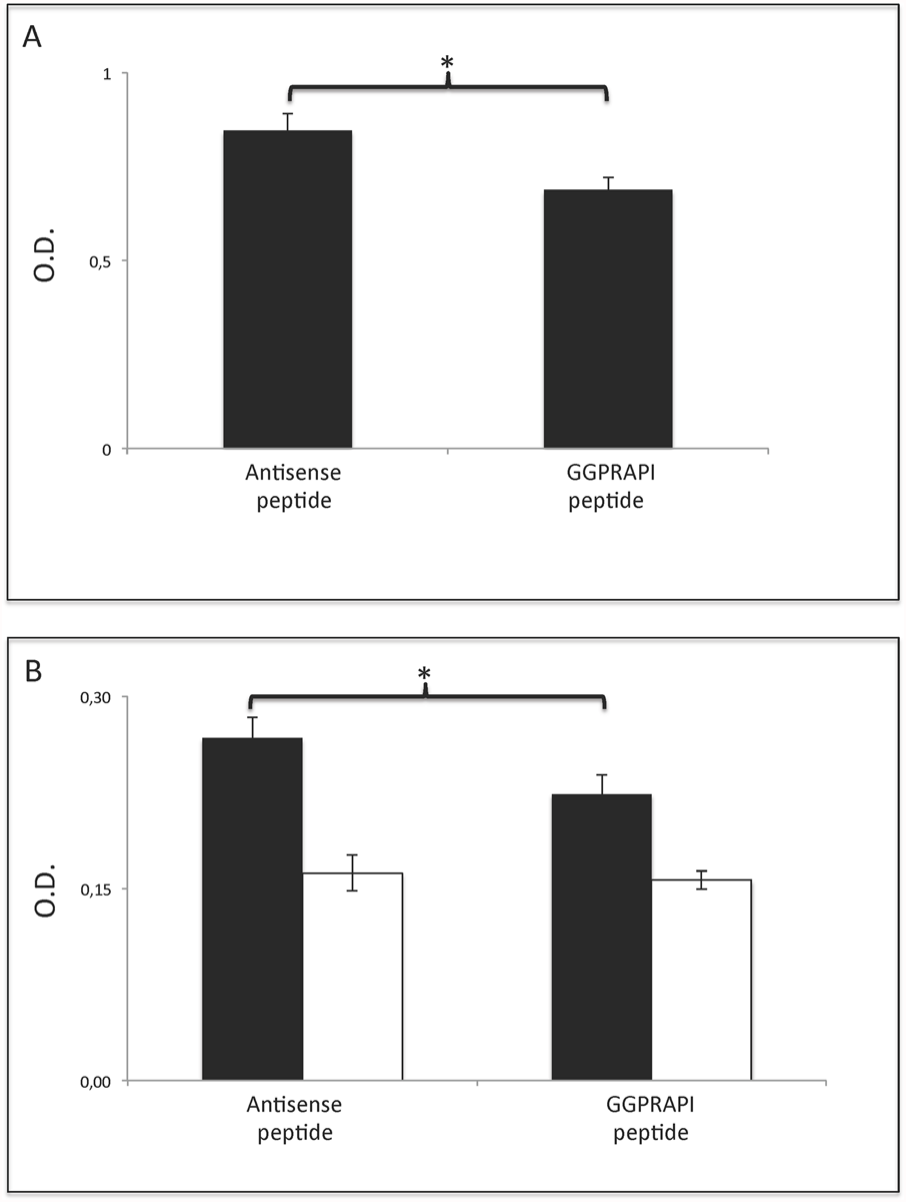
Ubiquitin is specifically recognized by the immunized rabbit antiserum and the index patient serum as determined by binding inhibition. A) Addition of 600μg GPGRAPI peptide (p<0,01), but not a control peptide significantly decreases binding of the immunized serum to Ubiquitin. B) Similarly, serum from the index patient (black columns) was competitively inhibited. A human control serum (white columns) bound lower than the index patient serum and was not inhibited by GPGRAPI peptide. Error bars represent standard deviation of triplicates, * indicates p <0.01.

The 36kD band to band revealed glyceraldehyde-dehydrogenase (GAP-DH) as the dominant content (57.9%). Other contents were annexin A2 (12.1%) and additional 24 proteins well below 4% content. Next, we investigated whether GAP-DH and ubiquitin are corresponding antigens to the peptide sequence GPGRAPI. Binding of both polyclonal rabbit and human serum to GAP-DH were shown to be non-specific (data not shown). In contrast, binding of polyclonal rabbit antiserum (Fig. 4A) and serum from the index patient (Fig. 4B) to ubiquitin was inhibited competitively by a synthetic GPGRAPI peptide.

Our data demonstrate that the antibody response of the index patient is directed against a sequence similar to GPGRAPI within ubiquitin. Upon sequence comparison, ubiquitin and the selected peptide sequence GPGRAPI showed 1 identical amino acid and 3 of similar physico-chemical properties at position 45 to 51 of ubiquitin (Table 2). In comparison with the other selected motifs the dominant similarity seems to be GRAxI.

**Table 2:** 

Ubiquitin	41-	Q	R	l	L	I	F	A	G	K	Q	L	E	D	G	R	T	L	S	D	Y	N	I	-60	
Selected motif						1-	G	P	G	R	A	P	I	-7											
Phage clones							G	P	G	R	A	P	I												(7X)
									G	R	A	S	I	W	P										(1X)
							G	M	G	R	E	S	A												(6X)
								V	G	R	R	P	W	F											(5X)

### Humoral immune response against ubiquitin

We next investigated a patient population undergoing cystoscopic bladder tumor resection for suspected TCC or undergoing cystectomy for proven TCC for antibody response against ubiquitin (n = 57). All cases were primary tumors and without previous transurethral operations/resections or vaccinations within one year before operation. Serum samples from healthy donors (n = 16) and prostate cancer (n = 8) patients served as negative controls. A positive immune reaction was defined as a twofold higher value of healthy donors immune response.

**Fig 5:**
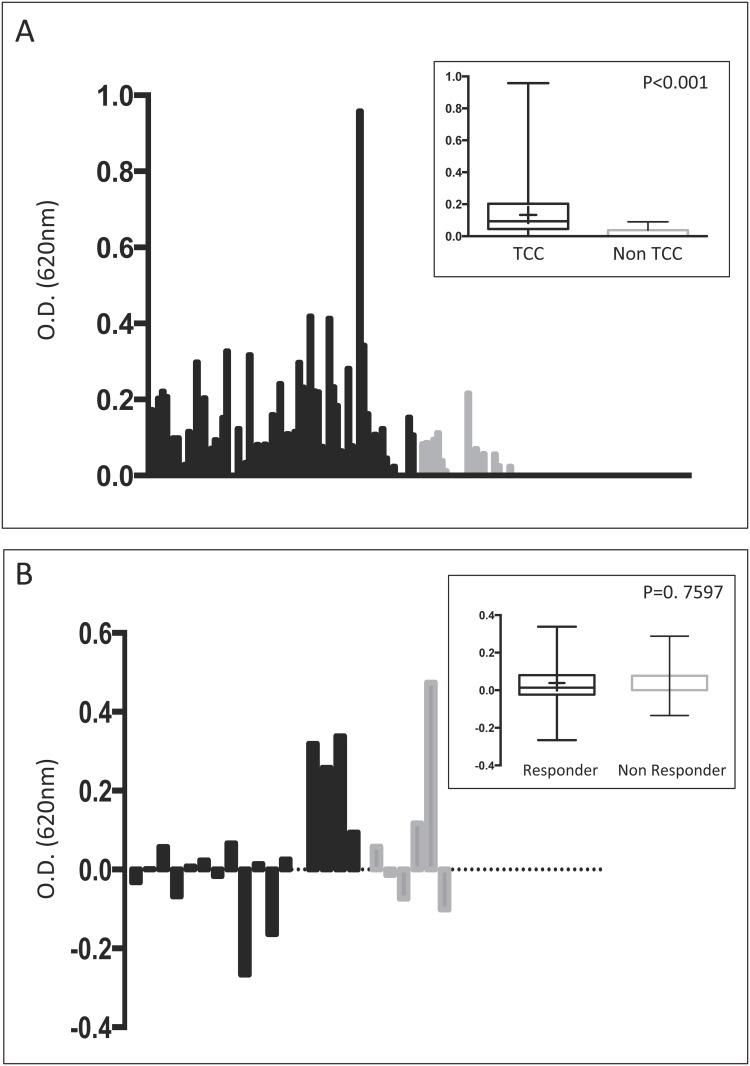
ELISA analysis of anti Ubiquitin titers. Left axis depicts absorbance/optical density (O.D.) at 620nm. A) TCC patients (black columns) have significant higher titers compared to non TCC patients (grey columns) (p<0.001). B) Titers from patients undergoing BCG immunotherapy for high risk TCC do not differ when comparing responder (black columns) to non responder (white columns). Bars represent titer changes against Ubiquitin during BCG-therapy. Inlets depict results of Mann Whitney U test (box and whiskers plots with maximum and minimum range).

Patients with TCC had significantly higher antibody titers against ubiquitin than healthy donors (p<0.007), prostate cancer patients (p<0.0007) and all patients without tumor (p<0.0001) (Fig. 5). Titers from superficial tumors versus muscle invasive tumors as well as titers from low grade versus high grade tumors were not significantly different (p = 0.0929 and p = 0.2631, respectively. For antibody response against ubiquitin, sensitivity for detection of TCC was 0.44, specificity 0.96, positive predictive value 0.96 and negative predictive value 0.41.

Next, we investigated wether immunotherapy with Mycobacterium bovis Bacillus-Calmette-Guerin (BCG), which is the standard therapy for high risk TCC and well known to affect the humoral immune response, also influences ubiquitin titers. We analyzed ubiquitin antibody titers before and after BCG induction therapy in 48 patients. In all patients no previous BCG immunotherapy or vaccination against tuberculosis within 10years before had been performed. All cases TCC were first diagnoses. Induction treatment started 6–8 weeks after initial transurethral resection. Changes of titers are given in Fig. 5B and varied between patients between an increase, decrease or unchanged. Between the responding and non responding groups no significant difference was observed (p = 0.7597).

## Discussion

Since their first description in 1966 by Baldwin et al. [22] autoantibodies and the related immune response have increasingly moved into the focus of research on tumors including human bladder cancer and the immune response during a BCG-immunotherapy [15–18, 23, 24]. While circulating tumor antigens are too rare to currently be of diagnostic value, autoantibodies resemble an early and amplified response against tumor antigens [25]. Furthermore, antibodies can be easily measured and promise diagnostic value since immune tolerance is relatively robust.

In this report we used an open antibody fingerprinting approach [15] to evaluate the humoral immune response by combinatorial selection of a random peptide phage display library on IgG isolated from an index patient with high risk TCC. In contrast to previous studies, which limited the response to a given subset of antigens [16, 26], we herewith probed for the entire range of possible target antigens, including neoantigens. Indeed, humoral anti-tumor immune responses have been previously reported [17, 18, 27]. Our cohort of patients with TCC exhibit significant titers in 44% of cases with a high specificity and positive predictive value. To our knowledge, this is the first report demonstrating autoantibodies directed against ubiquitin in the context of cancer and in TCC of the bladder.

The Ubiquitin Proteasome System is an emerging field of research [11]. Intracellular protein homeostasis is maintained by degrading excess, mutated or misfolded proteins by poly-ubiquitination and successive cleavage by the proteasome. These protein fragments are then degraded to recycle ubiquitin and enable either single amino acids or smaller fragments to be presented by the MHC. Furthermore, upon cellular stress, such as lack of oxygen in quickly proliferating tumors, an increased amount of ubiqitinated proteins accumulates. Such an abnormal accumulation of ubiquitin or ubiquitinated proteins has been described in Parkinson’s disease, Alzheimer’s disease, as well as cancer such as B-CLL [12–14]. Given the well-known involvement of the immune system in bladder cancer it is plausible that ubiquitin may become an important target [17, 18].

Upon comparison of the selected peptide to ubiquitin using the Clustal W software (http://www.ebi.ac.uk/Tools/msa/clustalw2/) surprisingly only a moderate similarity was observed: one amino acid was identical and three more exchangeable due to similar physico-chemical properties (position 45–51) [28]. This could possibly indicate that ubiquitin is mutated in TCC. So far only one mutation of ubiquitin has been reported in the literature, the UBB+1 mutant, caused by a frameshift mutation [29], which is different to the peptide we selected here.

An alternative explanation for antibody production against ubiquitin in TCC may be that ubiquitin is involved in peptide degradation for antigen presentation. Normally, ubiquitin is cleaved from the degraded protein prior to antigen presentation. However, due to the high cell turnover in cancer, immunogenic peptides may be released still attached to ubiquitin, thereby inducing a specific immune response, e.g. via dendritic cells that endocytose extracellular material.

Several autoantibodies have already been reported in bladder cancer, including the heat shock protein (Hsp) 65 in the context of BCG immunotherapy [17, 18, 27, 30]. BCG immunotherapy has been shown to induce antibodies against Hsp65, which are of diagnostic importance [30]. We therefore investigated whether BCG therapy induced a humoral immune response against ubiquitin. However, no significant change of titers for antibody production against ubiquitin or GAP-DH was noted after BCG therapy, indicating that this humoral target is not a focus during BCG therapy. This finding is in concordance with one of the current hypotheses that BCG induces a strong antitumoral immune response by molecular mimicry between exogenously administered BCG and the tumor cells [30]. However, as prokaryotes such as BCG do not contain ubiquitin or the ubiquitin proteasome system per se, such a molecular mimicry is not possible.

Immunotherapy in bladder cancer is a well established treatment option. *Mycobacterium bovis* BCG induces a strong immune response, eliminating the tumor in about 70% of cases. However, strong side effects are observed and several attempts have been undertaken to find safer alternatives. Mostly, these involve modifications of BCG, e.g. IL-2 secreting BCG, but also alternative agents such as Keyhole Limpet hemocyanin [31, 32]. So far all attempts to replace the current BCG- therapy regimen have had little effect, which may be due to the fact that none of them used immunologic targets. In our study we aimed to determine an immunologically relevant target by fingerprobing the patients own antibody response. Such targets promise to be of high value for any immunotherapy. An anti-ubiquitin directed immunotherapy might offer the basis for an effective alternative immunotherapy.

The limits of this study are the yet small numbers of pathological circumstances in which anti-ubiquitin autoantibodies are measured. Further clinical studies, e.g. in context of infection or further, non-urinary cancers are needed. Although the identification of new diagnostic biomarkers was one of our original intent, the here described antibody production seems unlikely to be of diagnostic value due to low sensitivity. Nevertheless, based on the observation that 44% of cases with TCC exhibit significant titers with high specificity and positive predictive value we suggest that ubiquitin is an interesting candidate for further diagnostic and possibly immune modulating studies in the future.

## Conclusion

This report is the first to demonstrate that ubiquitin is a target of the humoral immune system in bladder cancer. Even though sensitivity is relatively low, high specificity makes this protein an interesting candidate for diagnostic purposes, and possibly even for future vaccination strategies.
